# Crystal structure of (*E*)-*N*′-(3,4-di­hydroxy­benzyl­idene)-4-hy­droxy­benzohydrazide^1^

**DOI:** 10.1107/S2056989019010442

**Published:** 2019-07-28

**Authors:** Suchada Chantrapromma, Huey Chong Kwong, Patcharawadee Prachumrat, Thawanrat Kobkeatthawin, Tze Shyang Chia, Ching Kheng Quah

**Affiliations:** a Department of Chemistry, Faculty of Science, Prince of Songkla University, Hat-Yai, Songkhla 90112, Thailand; b Department of Chemistry, Faculty of Science, Universiti Putra Malaysia, 43400 UPM Serdang, Selangor Darul Ehsan, Malaysia; c X-ray Crystallography Unit, School of Physics, Universiti Sains Malaysia, 11800 USM, Penang, Malaysia

**Keywords:** crystal structure, hydrazide, mol­ecular conformation, anti­oxidant, α-glucosidase inhibitory

## Abstract

In the title benzohydrazide derivative, C_14_H_12_N_2_O_4_, the azomethine C=N double bond has an *E* configuration. The hydrazide connecting bridge, (C=O)—(NH)—N=(CH), is nearly planar with C—C—N—N and C—N—N=C torsion angles of −177.33 (10) and −174.98 (12)°, respectively. The 4-hy­droxy­phenyl and 3,4-di­hydroxy­phenyl rings are slightly twisted, making a dihedral angle of 9.18 (6)°. In the crystal, mol­ecules are connected by N—H⋯O and O—H⋯O hydrogen bonds into a three-dimensional network, while further consolidated *via* π–π inter­actions [centroid–centroid distances = 3.6480 (8) and 3.7607 (8) Å]. The conformation is compared to those of related benzyl­idene-4-hy­droxy­benzohydrazide derivatives.

## Abstract 2

The title compound is constructed by a 4-hy­droxy­benzyl ring, a 3,4-di­hydroxy­benzyl­idenyl ring and a hydrazide-connecting bridge. The overall conformation of the title compound are discussed and compared to the related structures. In the crystal, mol­ecules are connected by N—H⋯O, O—H⋯O and π–π inter­actions.

## Chemical context   

Hydrazides and hydrazones are important synthons for several transformations and have gained importance because of their various biological and clinical applications (Narasimhan *et al.*, 2010 ▸). Benzohydrazide derivatives containing an azomethine (–NHN=CH–) group have been reported to possess diverse biological activities such as anti­tumor (Xia *et al.*, 2007 ▸; Kumari & Bansal, 2018 ▸), anti­oxidant (Aziz *et al.*, 2014 ▸), anti­tubercular and anti­microbial (Maheswari & Manjula, 2015 ▸) and *α*-glucosidase inhibition (Taha *et al.*, 2015 ▸) activities. The inter­esting biological activities of benzohydrazides led us to synthesize several benzohydrazides to study their bioactivities (Fun *et al.*, 2011 ▸; Horkaew *et al.*, 2011 ▸; Chantrapromma *et al.*, 2016 ▸), including the title compound (I), which was found to exhibit anti­oxidant activity with an IC_50_ value of 0.035 ± 0.004 m*M* (ascorbic acid used as the reference standard; Thaipong *et al.*, 2006 ▸) and α-glucosidase inhibitory activity with an IC_50_ value of 0.014 ± 0.001 m*M* (acarbose as the reference standard; Bachhawat *et al.*, 2011 ▸).
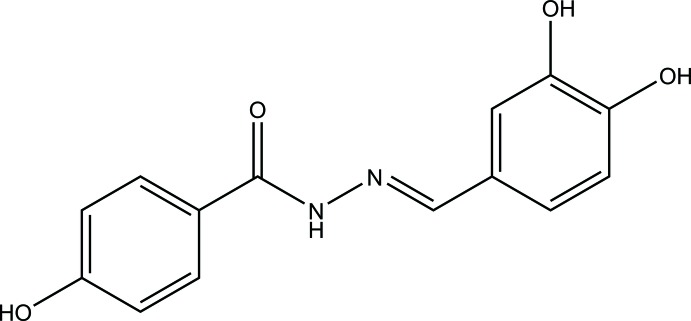


## Structural commentary   

**Figure 1:**
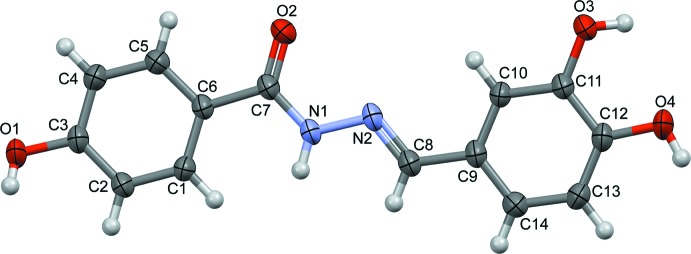
The mol­ecular structure of the title compound with the atom-labelling scheme and displacement ellipsoids at the 50% probability level.

The title hydrazide derivative, (I), consists of a 4-hy­droxy­phenyl ring, a 3,4-di­hydroxyphenyl ring and a hydrazide (C=O)—(NH)—N=(CH) connecting bridge (Fig. 1 ▸). The C6—C7, C7—N1 and C8—C9 bond lengths of 1.4861 (15), 1.3385 (17) and 1.4584 (16) Å, respectively, confirm their single-bond character, whereas the C7=O2 and N2=C8 bond lengths of 1.2403 (15) and 1.2738 (17) Å, respectively, confirm the presence of the double bonds. The *sp* ^2^-hybridized character of atoms C7 and C8 is further supported by the bond angles C6—C7—N1 [116.49 (11)°] and N2—C8—C9 [120.86 (12)°]. The bond lengths and angles of the central hydrazide connecting bridge are consistent with those in related structures (Fun *et al.*, 2011 ▸; Chantrapromma *et al.*, 2016 ▸). The mol­ecule exhibits an *E* configuration with respect to the azomethine C=N double bond. As the torsion angle C6—C7—N1—N2 [−177.33 (10)°] and C7—N1—N2—C8 [−174.98 (12)°] are both in an *anti-periplanar* conformation, the overall conformation for the hydrazide connecting bridge is almost planar. Furthermore, the 4-hy­droxy­phenyl and 3,4-di­hydroxy­phenyl rings are also coplanar to the corresponding azomethine and carbonyl double bonds, with torsion angles N2—C8—C9—C10 [−0.76 (19)°] and C5—C6—C7—O2 [−1.18 (19)°] both in a *syn-periplanar* conformation. Those torsion angles result in an overall flat shape of the title compound with the dihedral angle between the terminal benzene rings being 9.18 (6)°.

## Supra­molecular features   

**Figure 2:**
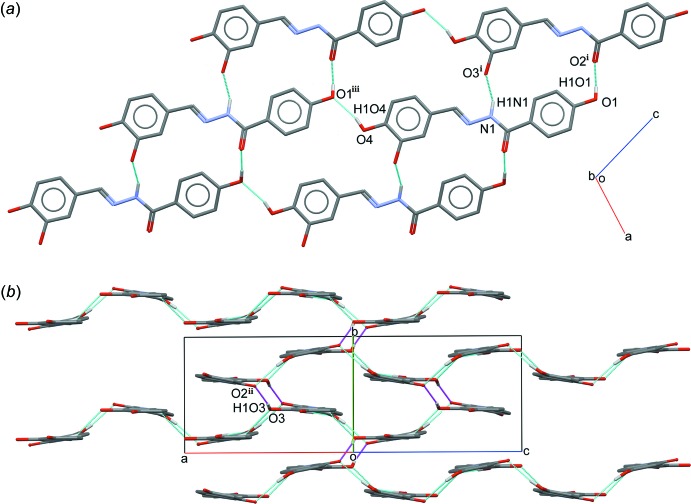
(*a*) A partial packing diagram of the title compound, showing a two-dimensional plate formed by O—H⋯O and N—H⋯O hydrogen bonds (cyan dotted lines). [Symmetry codes: (i) *x*, −*y* + 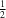, *z* + 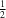; (iii) *x* − 1, *y*, *z* − 1.] (*b*) A partial packing diagram of the title compound with additional O—H⋯O hydrogen bonds (magenta dotted lines). [Symmetry code: (ii) −*x* + 1, −*y* + 1, −*z*.] Hydrogen atoms not involved in with these inter­actions are omitted for clarity.

In the crystal, mol­ecules are linked by N—H⋯O and O—H⋯O hydrogen bonds (Table 1 ▸) into a three-dimensional network. Mol­ecules are connected into infinite chains along [101] through an O4—H1*O*4⋯O1^iii^ hydrogen bond and those chains are further connected into two-dimensional plates parallel to the *ac* plane *via* N1—H1*N*1⋯O3^i^ and O1—H1*O*1⋯O2^i^ hydrogen bonds with an 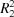
(18) ring motif (Fig. 2*a* ▸ and 3*a* ▸; symmetry codes as in Table 1 ▸). Those plate are inter­connected *via* an O3—H1*O*3⋯O2^ii^ hydrogen bond with an 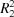
(20) ring motif, forming a three-dimensional network (Fig. 2*b* ▸ and 3*b* ▸; symmetry code as in Table 1 ▸). In addition, the mol­ecules are further stabilized by π–π inter­actions involving both aromatic rings with *Cg*1⋯*Cg*2^iv^ = 3.6480 (8) Å and *Cg*1⋯*Cg*2^v^ = 3.7607 (8) Å [symmetry codes: (iv) 1 − *x*, −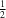 + *y*, 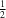 − *z*; (v) 1 − *x*, 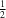 + *y*, 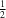 − *z*; *Cg*1 and *Cg*2 are the centroids of the C1–C6 and C9–C14 aromatic rings, respectively.]

## Database survey   

**Figure 4:**
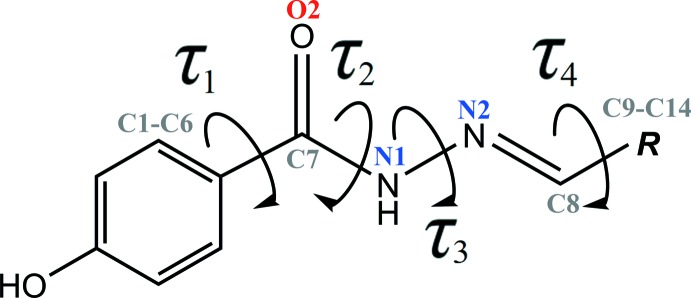
General chemical diagram, showing torsion angles *τ*_1_, *τ*_2_, *τ*_3_ and *τ*_4_ in the benzyl­idene-4-hy­droxy­benzohydrazide derivative.

A search of the Cambridge Structural Database (CSD version 5.40, last update May 2019; Groom *et al.*, 2016 ▸) using (*E*)-*N*′-benzenyl­idene-4-hy­droxy­benzohydrazide as the reference moiety resulted in 31 structures with different substituents at the benzyl­idenyl ring. The different substituent (***R***) together with selected torsion angles, *τ*_1_ (C5—C6—C7—O2), *τ*_2_ (C6—C7—N1—N2), *τ*_3_ (C7—N1—N2—C8) and *τ*_4_ (N2—C8—C9—C10) as shown in Fig. 4 ▸, and the dihedral angle between the terminal aromatic rings are summarized in Table 2 ▸. The torsion angles *τ*_2_ and *τ*_3_ are *anti-periplanar* (151.7–179.8°), showing that the hydrazide connecting bridges are nearly planar. As for the torsion angle *τ*_4_, all structures adopt a *syn-periplanar* conformation (0.6–19.6°). Similar to the title compound, the *τ*_1_ torsion angles for most of the structures are *syn-periplanar* (2.0–29.1°). However, there are three outliers (CEDBAQ, HUCWOS and PAQJID) whose *τ*_1_ torsion angles are *syn-clinal* (34.9–50.9°). By comparing the dihedral angles, the structures can be divided into planar compounds (dihedral angle = 2.5–29.3°) and non-planar compounds (dihedral angle = 30.5–77.3°). In general, as the hydrazide-connecting bridges are nearly planar, relatively flat *τ*_1_ and *τ*_4_ torsion angles are observed in the former compounds, while relatively twisted *τ*_1_ and *τ*_4_ torsion angles are observed in the latter.

## Synthesis and crystallization   

The title compound (I) was prepared by dissolving 4-hy­droxy­benzohydrazide (2 mmol, 0.30 g) in ethanol (10 ml). A solution of 3,4-di­hydroxy­benzaldehyde (2 mmol, 0.28 g) in ethanol (10 ml) was then added to the reaction. The mixture was refluxed for 6 h and the white solid of the product that appeared was collected by filtration, washed with ethanol and dried in air. Colourless single crystals of (I) were obtained after recrystallization from methanol at room temperature for several days.

M.p. 572–573 K. UV–Vis (MeOH) λ_max_ 213, 327 nm; FT–IR (KBr) ν (cm^−1^): 3121 (O—H stretching), 2800 (C—H aromatic stretching), 1615 (amide C=O stretching), 1570 (C=N stretching), 1506 (C=C stretching of aromatic compound) cm^−1^; ^1^H NMR (300 MHz, *d*_6_-DMSO) *δ* 11.39 (*s*, 1H, NH), 10.10 (*s*, 1H, Ar—OH), 8.23 (*s*, 1H, N=CH), 7.77 (*d*, *J* = 8.4 Hz, 2H, Ar—H), 6.84 (*d*, *J* = 8.4, 2H, Ar—H), 9.33 (*s*, 2H, Ar—OH), 7.22 (*s*, 1H, Ar—H), 6.90 (*d*, *J* = 7.8 Hz, 1H, Ar—H), 6.77 (*d*, *J* = 8.1 Hz, 1H, Ar—H).

## Refinement   

Crystal data, data collection and structure refinement details are summarized in Table 3 ▸ *.* C*-*bound H atoms were positioned geometrically (C*—*H *=* 0*.*93 Å) and refined using a riding model with *U*_iso_(H) = 1.2*U*_eq_(C). All O- and N-bound H atoms were located in a difference-Fourier map and refined freely [O—H = 0.80 (2)–0.88 (2) Å and N—H = 0.87 (2) Å].

## Supplementary Material

Crystal structure: contains datablock(s) I. DOI: 10.1107/S2056989019010442/is5518sup1.cif

Structure factors: contains datablock(s) I. DOI: 10.1107/S2056989019010442/is5518Isup2.hkl

Supporting information file. DOI: 10.1107/S2056989019010442/is5518Isup3.cml

CCDC reference: 1942396

Additional supporting information:  crystallographic information; 3D view; checkCIF report

## Figures and Tables

**Figure 3:**
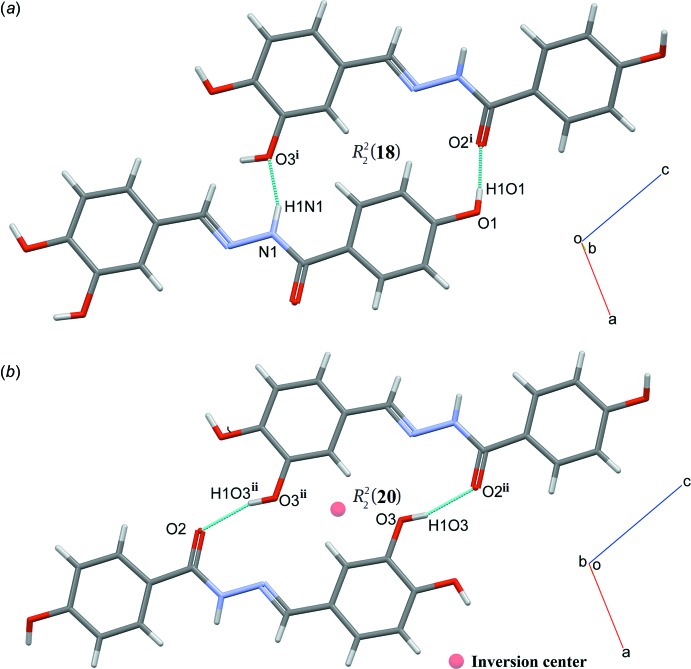
A view of dimers with (*a*) 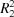
(18) and (*b*) 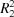
(20) ring motifs. [Symmetry codes: (i) *x*, −*y* + 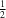, *z* + 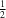; (ii) −*x* + 1, −*y* + 1, −*z*.]

**Table 1:** Hydrogen-bond geometry (Å, °)

*D*—H⋯*A*	*D*—H	H⋯*A*	*D*⋯*A*	*D*—H⋯*A*
O1—H1*O*1⋯O2^i^	0.80 (2)	1.92 (2)	2.7203 (15)	171 (2)
O3—H1*O*3⋯O2^ii^	0.88 (2)	2.17 (2)	3.0276 (13)	163 (2)
O4—H1*O*4⋯O1^iii^	0.82 (2)	1.93 (2)	2.7379 (16)	166 (2)
N1—H1*N*1⋯O3^i^	0.87 (2)	2.24 (2)	3.0017 (16)	146.1 (19)

Symmetry codes: (i) 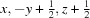; (ii) 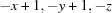; (iii) 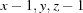.

**Table 2:** Selected torsion angles (°) and the dihedral angle (°) between the terminal benzene rings

Compound	***R***	*τ*_1_	*τ*_2_	*τ*_3_	*τ*_4_	Dihedral angle
Planar						
(I)	3,4-di­hydroxy­phen­yl	−1.2	−177.3	−175.0	−0.8	9.2
ABALIA (Fun *et al.*, 2011 ▸)	3-hy­droxy-4-meth­oxy­phen­yl	3.2	178.4	170.1	−14.2	24.2
CECZOB (Subashini *et al.*, 2012 ▸)	4-chloro­phen­yl	26.1	−174.4	166.6	−8.9	5.8
CECZUH (Subashini *et al.*, 2012 ▸)	4-bromo­phen­yl	25.6	−174.9	169.0	−7.2	9.8
ESOTUD (Chantrapromma *et al.*, 2016 ▸)	3-meth­oxy­phen­yl	−19.4, 20.7	−173.5, −177.8	−175.7, −173.0	1.2, 0.6	24.0, 29.3
HOZBII (Li & Ban, 2009 ▸)	4-nitro­phen­yl	2.0	177.7	178.3	−0.6	2.5
IJUKEE (Zhang, 2011 ▸)	4-hy­droxy-3-nitro­phen­yl	−7.2	−177.0	−179.3	6.0	5.5
IRAXEF (Sánchez-Lozano *et al.*, 2011 ▸)	2,4-di­hydroxy­phen­yl	−7.7	−177.8	−177.2	−4.1	6.9
MOZPEX (Ren, 2009 ▸)	3,5-di­chloro-2-hy­droxy­phen­yl	12.3	178.7	−179.4	−7.3	5.1
ROFMOP (Xue *et al.*, 2008 ▸)	3-bromo-5-chloro-2-hy­droxy­phen­yl	−2.3	175.9	−176.5	−1.3	3.0
TEWLAL (Ayyannan *et al.*, 2016 ▸)	5-bromo-2-hy­droxy­phen­yl	−15.7	−173.6	168.9	3.1	27.0
WACVON (Shalash *et al.*, 2010 ▸)	4-hy­droxy-3-meth­oxy­phen­yl	−34.2	−175.5	174.7	15.4	28.6
WACXOP (Huang, 2010 ▸)	2,4-di­chloro­phen­yl	−14.3	−179.8	−175.0	3.0	7.0
YAGYAI (Horkaew *et al.*, 2011 ▸)	3,4,5-tri­meth­oxy­phen­yl	−10.6	172.2	175.8	2.8	19.4
YIFPAF (Salhin *et al.*, 2007 ▸)	2-hy­droxy­phen­yl	18.8	179.5	178.7	3.3	21.7
ZAPKOS (Hou, 2012 ▸)	3-nitro­phen­yl	−14.6	169.4	177.4	13.8	9.2
ZIPLAO (Prachumrat *et al.*, 2018 ▸)	2,3-di­meth­oxy­phen­yl	9.6	−175.3	172.9	−1.3	9.3
Non-planar						
CABWUA (Meng *et al.*, 2014 ▸)	2-hy­droxy-5-methyl­phen­yl	18.4	−178.5	−169.8	8.0	40.8
CEDBAQ (Subashini *et al.*, 2012 ▸)	4-(di­ethyl­amino)­phen­yl	34.9	−178.5	−151.7	8.75	77.3
HUCVIL (Hao, 2009 ▸)	2-chloro­phen­yl	−22.5	−179.2	177.4	−4.2	30.5
HUCWOS (Shi, 2009 ▸)	4-meth­oxy­phen­yl	−50.9	−177.5	174.8	9.2	46.6
MOSPEQ (Qiu, 2009 ▸)	5-chloro-2-hy­droxy­phen­yl	19.0	−178.5	−170.9	7.59	40.2
PAQJID (Gopal Reddy *et al.*, 2017 ▸)	4-ethyl­phen­yl	−39.9	171.1	173.9	7.4	49.9
PAWVUG (Rassem *et al.*, 2012*a* ▸)	2-meth­oxy­phen­yl	29.1	−166.8	−175.1	19.2	66.6
PEDGOW (Saad *et al.*, 2012 ▸)	3-chloro­phen­yl	−21.1	179.5	175.3	−9.3	39.0
XEBYUA (Rassem *et al.*, 2012*b* ▸)	2-hy­droxy-4-meth­oxy­phen­yl	28.7	178.1	−169.8	1.3	40.6

**Table 3:** Experimental details

Crystal data	
Chemical formula	C_14_H_12_N_2_O_4_
*M*_r_	272.26
Crystal system, space group	Monoclinic, *P*2_1_/*c*
Temperature (K)	296
*a*, *b*, *c* (Å)	11.5352 (8), 7.1711 (5), 15.0606 (10)
β (°)	108.548 (2)
*V* (Å^3^)	1181.10 (14)
*Z*	4
Radiation type	Mo *K*α
μ (mm^−1^)	0.11
Crystal size (mm)	0.80 × 0.21 × 0.07

Data collection	
Diffractometer	Bruker APEXII DUO CCD area-detector
Absorption correction	Multi-scan (*SADABS*; Bruker, 2012 ▸)
*T*_min_, *T*_max_	0.924, 0.954
No. of measured, independent and observed [*I* > 2σ(*I*)] reflections	22559, 3200, 2453
*R*_int_	0.024
(sin θ/λ)_max_ (Å^−1^)	0.686

Refinement	
*R*[*F* ^2^ > 2σ(*F* ^2^)], *wR*(*F* ^2^), *S*	0.050, 0.161, 1.05
No. of reflections	3200
No. of parameters	197
H-atom treatment	H atoms treated by a mixture of independent and constrained refinement
Δρ_max_, Δρ_min_ (e Å^−3^)	0.35, −0.19

Computer programs: *APEX2* and *SAINT* (Bruker, 2012 ▸), *SHELXS97* (Sheldrick, 2008 ▸), *SHELXL2013* (Sheldrick, 2015 ▸), *Mercury* (Macrae *et al.*, 2006 ▸) and *PLATON* (Spek, 2009 ▸).

## supplementary crystallographic information

### Crystal data

**Table:**

C_14_H_12_N_2_O_4_	*F*(000) = 568
*M**_r_* = 272.26	*D*_x_ = 1.531 Mg m^−^^3^
Monoclinic, *P*2_1_/*c*	Mo *K*α radiation, λ = 0.71073 Å
*a* = 11.5352 (8) Å	Cell parameters from 6293 reflections
*b* = 7.1711 (5) Å	θ = 2.9–29.2°
*c* = 15.0606 (10) Å	µ = 0.11 mm^−^^1^
β = 108.548 (2)°	*T* = 296 K
*V* = 1181.10 (14) Å^3^	Plate, colourless
*Z* = 4	0.80 × 0.21 × 0.07 mm

### Data collection

**Table:**

Bruker APEXII DUO CCD area-detector diffractometer	3200 independent reflections
Radiation source: fine-focus sealed tube	2453 reflections with *I* > 2σ(*I*)
Graphite monochromator	*R*_int_ = 0.024
φ and ω scans	θ_max_ = 29.2°, θ_min_ = 1.9°
Absorption correction: multi-scan (SADABS; Bruker, 2012)	*h* = −15→15
*T*_min_ = 0.924, *T*_max_ = 0.954	*k* = −9→9
22559 measured reflections	*l* = −20→20

### Refinement

**Table:**

Refinement on *F*^2^	0 restraints
Least-squares matrix: full	Hydrogen site location: mixed
*R*[*F*^2^ > 2σ(*F*^2^)] = 0.050	H atoms treated by a mixture of independent and constrained refinement
*wR*(*F*^2^) = 0.161	*w* = 1/[σ^2^(*F*_o_^2^) + (0.0999*P*)^2^ + 0.1317*P*] where *P* = (*F*_o_^2^ + 2*F*_c_^2^)/3
*S* = 1.05	(Δ/σ)_max_ < 0.001
3200 reflections	Δρ_max_ = 0.35 e Å^−^^3^
197 parameters	Δρ_min_ = −0.18 e Å^−^^3^

### Special details

**Table:**

Geometry. All esds (except the esd in the dihedral angle between two l.s. planes) are estimated using the full covariance matrix. The cell esds are taken into account individually in the estimation of esds in distances, angles and torsion angles; correlations between esds in cell parameters are only used when they are defined by crystal symmetry. An approximate (isotropic) treatment of cell esds is used for estimating esds involving l.s. planes.

### Fractional atomic coordinates and isotropic or equivalent isotropic displacement parameters (Å^2^)

**Table:**

	*x*	*y*	*z*	*U*_iso_*/*U*_eq_	
O1	0.87093 (9)	0.31797 (18)	0.75119 (6)	0.0493 (3)	
O2	0.72990 (8)	0.42338 (15)	0.31133 (6)	0.0439 (3)	
O3	0.36583 (9)	0.37514 (15)	−0.12560 (6)	0.0432 (3)	
O4	0.11997 (10)	0.35694 (18)	−0.18715 (7)	0.0547 (3)	
N1	0.54178 (10)	0.37659 (17)	0.32223 (7)	0.0392 (3)	
N2	0.48862 (10)	0.38068 (17)	0.22612 (7)	0.0387 (3)	
C1	0.64041 (11)	0.3493 (2)	0.52044 (8)	0.0360 (3)	
H1A	0.555998	0.341901	0.493279	0.043*	
C2	0.69125 (11)	0.32969 (19)	0.61626 (8)	0.0370 (3)	
H2A	0.641478	0.308993	0.653221	0.044*	
C3	0.81673 (12)	0.34105 (19)	0.65681 (8)	0.0359 (3)	
C4	0.89049 (12)	0.3773 (2)	0.60204 (9)	0.0429 (3)	
H4A	0.974622	0.389056	0.629625	0.051*	
C5	0.83860 (12)	0.3960 (2)	0.50639 (9)	0.0398 (3)	
H5A	0.888474	0.419686	0.469812	0.048*	
C6	0.71320 (11)	0.38002 (16)	0.46381 (8)	0.0312 (3)	
C7	0.66306 (11)	0.39454 (18)	0.36014 (8)	0.0334 (3)	
C8	0.37218 (12)	0.37651 (18)	0.19709 (8)	0.0357 (3)	
H8A	0.329660	0.374477	0.240199	0.043*	
C9	0.30468 (12)	0.37489 (17)	0.09713 (8)	0.0332 (3)	
C10	0.36617 (11)	0.38007 (18)	0.03124 (8)	0.0339 (3)	
H10A	0.451058	0.387612	0.051040	0.041*	
C11	0.30251 (11)	0.37414 (17)	−0.06263 (8)	0.0332 (3)	
C12	0.17505 (12)	0.36368 (19)	−0.09316 (9)	0.0377 (3)	
C13	0.11375 (12)	0.3624 (2)	−0.02790 (9)	0.0434 (3)	
H13A	0.028756	0.358471	−0.047790	0.052*	
C14	0.17809 (12)	0.3669 (2)	0.06705 (9)	0.0400 (3)	
H14A	0.136252	0.364586	0.110600	0.048*	
H1O1	0.8288 (18)	0.256 (3)	0.7732 (15)	0.074 (6)*	
H1O3	0.324 (2)	0.421 (3)	−0.1808 (16)	0.092 (7)*	
H1O4	0.046 (2)	0.345 (3)	−0.1964 (16)	0.085 (7)*	
H1N1	0.4954 (19)	0.342 (3)	0.3546 (14)	0.069 (6)*	

### Atomic displacement parameters (Å^2^)

**Table:**

	*U*^11^	*U*^22^	*U*^33^	*U*^12^	*U*^13^	*U*^23^
O1	0.0364 (5)	0.0836 (8)	0.0215 (4)	−0.0105 (5)	0.0004 (4)	0.0057 (4)
O2	0.0376 (5)	0.0700 (7)	0.0267 (4)	−0.0036 (4)	0.0139 (4)	−0.0016 (4)
O3	0.0344 (5)	0.0719 (7)	0.0236 (4)	0.0037 (4)	0.0095 (4)	0.0026 (4)
O4	0.0352 (6)	0.0991 (9)	0.0227 (5)	−0.0083 (5)	−0.0009 (4)	0.0033 (5)
N1	0.0326 (6)	0.0651 (7)	0.0185 (5)	−0.0038 (5)	0.0060 (4)	0.0023 (4)
N2	0.0380 (6)	0.0576 (7)	0.0182 (5)	−0.0033 (5)	0.0056 (4)	0.0010 (4)
C1	0.0254 (6)	0.0554 (7)	0.0248 (6)	−0.0019 (5)	0.0046 (4)	−0.0004 (5)
C2	0.0319 (6)	0.0558 (8)	0.0232 (6)	−0.0014 (5)	0.0086 (5)	0.0005 (5)
C3	0.0335 (6)	0.0485 (7)	0.0220 (5)	−0.0024 (5)	0.0037 (5)	−0.0001 (5)
C4	0.0272 (6)	0.0688 (9)	0.0289 (6)	−0.0039 (6)	0.0037 (5)	0.0022 (6)
C5	0.0307 (6)	0.0611 (8)	0.0284 (6)	−0.0029 (5)	0.0105 (5)	0.0014 (5)
C6	0.0300 (6)	0.0411 (6)	0.0213 (5)	0.0007 (4)	0.0063 (4)	−0.0010 (4)
C7	0.0337 (6)	0.0440 (7)	0.0222 (6)	−0.0001 (5)	0.0086 (5)	−0.0019 (4)
C8	0.0352 (7)	0.0481 (7)	0.0231 (6)	0.0003 (5)	0.0081 (5)	0.0012 (5)
C9	0.0332 (6)	0.0416 (6)	0.0219 (5)	0.0005 (5)	0.0046 (5)	0.0009 (4)
C10	0.0261 (5)	0.0492 (7)	0.0234 (6)	0.0004 (5)	0.0038 (4)	−0.0001 (5)
C11	0.0311 (6)	0.0443 (7)	0.0234 (5)	0.0008 (5)	0.0073 (4)	0.0010 (4)
C12	0.0313 (6)	0.0521 (7)	0.0243 (6)	−0.0013 (5)	0.0012 (5)	0.0026 (5)
C13	0.0255 (6)	0.0686 (9)	0.0321 (7)	−0.0003 (5)	0.0038 (5)	0.0046 (6)
C14	0.0329 (6)	0.0582 (8)	0.0298 (6)	0.0015 (5)	0.0110 (5)	0.0037 (5)

### Geometric parameters (Å, º)

**Table:**

O1—C3	1.3688 (14)	C4—C5	1.3794 (18)
O1—H1O1	0.80 (2)	C4—H4A	0.9300
O2—C7	1.2403 (15)	C5—C6	1.3880 (18)
O3—C11	1.3688 (15)	C5—H5A	0.9300
O3—H1O3	0.88 (2)	C6—C7	1.4861 (15)
O4—C12	1.3556 (15)	C8—C9	1.4584 (16)
O4—H1O4	0.83 (3)	C8—H8A	0.9300
N1—C7	1.3385 (17)	C9—C14	1.3856 (18)
N1—N2	1.3811 (14)	C9—C10	1.3918 (17)
N1—H1N1	0.87 (2)	C10—C11	1.3709 (16)
N2—C8	1.2738 (17)	C10—H10A	0.9300
C1—C2	1.3812 (16)	C11—C12	1.3959 (18)
C1—C6	1.3916 (17)	C12—C13	1.3815 (19)
C1—H1A	0.9300	C13—C14	1.3861 (17)
C2—C3	1.3828 (18)	C13—H13A	0.9300
C2—H2A	0.9300	C14—H14A	0.9300
C3—C4	1.3852 (19)		
			
C3—O1—H1O1	111.0 (15)	O2—C7—N1	121.71 (11)
C11—O3—H1O3	113.5 (15)	O2—C7—C6	121.79 (11)
C12—O4—H1O4	107.2 (16)	N1—C7—C6	116.49 (11)
C7—N1—N2	120.02 (11)	N2—C8—C9	120.86 (12)
C7—N1—H1N1	122.4 (13)	N2—C8—H8A	119.6
N2—N1—H1N1	116.6 (13)	C9—C8—H8A	119.6
C8—N2—N1	115.30 (11)	C14—C9—C10	119.41 (11)
C2—C1—C6	121.20 (11)	C14—C9—C8	119.93 (12)
C2—C1—H1A	119.4	C10—C9—C8	120.66 (11)
C6—C1—H1A	119.4	C11—C10—C9	120.47 (11)
C1—C2—C3	119.49 (11)	C11—C10—H10A	119.8
C1—C2—H2A	120.3	C9—C10—H10A	119.8
C3—C2—H2A	120.3	O3—C11—C10	119.04 (11)
O1—C3—C2	121.31 (12)	O3—C11—C12	120.68 (11)
O1—C3—C4	118.49 (11)	C10—C11—C12	120.26 (11)
C2—C3—C4	120.20 (11)	O4—C12—C13	124.53 (12)
C5—C4—C3	119.69 (12)	O4—C12—C11	116.14 (12)
C5—C4—H4A	120.2	C13—C12—C11	119.33 (11)
C3—C4—H4A	120.2	C12—C13—C14	120.43 (12)
C4—C5—C6	121.12 (12)	C12—C13—H13A	119.8
C4—C5—H5A	119.4	C14—C13—H13A	119.8
C6—C5—H5A	119.4	C9—C14—C13	120.07 (12)
C5—C6—C1	118.24 (11)	C9—C14—H14A	120.0
C5—C6—C7	118.67 (11)	C13—C14—H14A	120.0
C1—C6—C7	123.09 (11)		
			
C7—N1—N2—C8	−174.98 (12)	N1—N2—C8—C9	−178.29 (10)
C6—C1—C2—C3	0.1 (2)	N2—C8—C9—C14	178.83 (12)
C1—C2—C3—O1	−178.44 (12)	N2—C8—C9—C10	−0.76 (19)
C1—C2—C3—C4	1.9 (2)	C14—C9—C10—C11	−1.11 (18)
O1—C3—C4—C5	178.19 (13)	C8—C9—C10—C11	178.47 (11)
C2—C3—C4—C5	−2.1 (2)	C9—C10—C11—O3	−178.51 (12)
C3—C4—C5—C6	0.3 (2)	C9—C10—C11—C12	0.29 (19)
C4—C5—C6—C1	1.6 (2)	O3—C11—C12—O4	−0.71 (19)
C4—C5—C6—C7	−177.78 (12)	C10—C11—C12—O4	−179.50 (12)
C2—C1—C6—C5	−1.9 (2)	O3—C11—C12—C13	179.81 (13)
C2—C1—C6—C7	177.51 (12)	C10—C11—C12—C13	1.03 (19)
N2—N1—C7—O2	3.3 (2)	O4—C12—C13—C14	179.03 (13)
N2—N1—C7—C6	−177.33 (10)	C11—C12—C13—C14	−1.5 (2)
C5—C6—C7—O2	−1.18 (19)	C10—C9—C14—C13	0.60 (19)
C1—C6—C7—O2	179.44 (13)	C8—C9—C14—C13	−178.99 (12)
C5—C6—C7—N1	179.42 (12)	C12—C13—C14—C9	0.7 (2)
C1—C6—C7—N1	0.04 (18)		

### Hydrogen-bond geometry (Å, º)

**Table:**

*D*—H···*A*	*D*—H	H···*A*	*D*···*A*	*D*—H···*A*
O1—H1*O*1···O2^i^	0.80 (2)	1.92 (2)	2.7203 (15)	171 (2)
O3—H1*O*3···O2^ii^	0.88 (2)	2.17 (2)	3.0276 (13)	163 (2)
O4—H1*O*4···O1^iii^	0.82 (2)	1.93 (2)	2.7379 (16)	166 (2)
N1—H1*N*1···O3^i^	0.87 (2)	2.24 (2)	3.0017 (16)	146.1 (19)

Symmetry codes: (i) *x*, −*y*+1/2, *z*+1/2; (ii) −*x*+1, −*y*+1, −*z*; (iii) *x*−1, *y*, *z*−1.
